# No-touch methods of terminal cleaning in the intensive care unit: results from the first large randomized trial with patient-centred outcomes

**DOI:** 10.1186/s13054-017-1705-2

**Published:** 2017-07-30

**Authors:** Vincenzo Russotto, Andrea Cortegiani, Pasquale Iozzo, Santi Maurizio Raineri, Cesare Gregoretti, Antonino Giarratano

**Affiliations:** 1 Department of Biopathology and Medical Biotechnologies (DIBIMED), Section of Anesthesia Analgesia Intensive Care and Emergency. Policlinico P. Giaccone, University of Palermo, Via del Vespro 129, 90127 Palermo, Italy; 2 Intensive Care Unit, Policlinico Paolo Giaccone, University of Palermo, Via del Vespro 129, 90127 Palermo, Italy

**Keywords:** Terminal cleaning, Sepsis, ICU-acquired infections, Multidrug resistant organisms

Environmental contamination may play a major role in intensive care unit (ICU)-acquired infections, despite current terminal cleaning standards [1]. Anderson et al. [2] recently performed the first large randomized trial investigating a no-touch method of terminal cleaning with a patient-centred outcome, and provided more robust data on the role of environmental contamination for healthcare-associated infections. The authors evaluated three different enhanced terminal disinfection methods (ultraviolet, UV light, UV light plus bleach, and bleach) compared to the reference standard for prevention of transmission of multidrug resistant organisms (MDROs) and *Clostridium difficile* to patients exposed to a room whose prior occupant was either colonized or infected with a MDRO.

The addition of UV light to the reference disinfection strategy (based on quaternary ammonium) reduced the transmission of the targeted MDROs (methicillin-resistant *Staphylococcus aureus* and vancomycin-resistant enterococci) by 30%, while no significant difference was observed when UV light was compared to bleach or to the combination of bleach and UV. Moreover, the authors did not observe a difference in colonization and infection by *C. difficile* when UV light was added to bleach (the standard disinfection method for *C. difficile*). We agree with them that this would represent the minimum effect of the UV strategy and that, in a real-life scenario with considerably less cleaning compliance, the benefit from UV-based enhanced terminal cleaning would have been more incisive. Notably, killing of *C. difficile* spores appeared to be time- and dose-dependent in a previously published study [3].

ICU-acquired infections are a major health problem worldwide [4]. Environmental contamination may pose an even higher challenge in this setting due to a number of factors: a higher prevalence of colonization and infection by MDROs, understaffing, the presence of sophisticated equipment with high-touch surfaces and specific cleaning procedures [1]. The benefit of no-touch methods for terminal cleaning may be theoretically higher in these circumstances. Unfortunately, the study did not provide data on *Acinetobacter* since only one exposed patient acquired this organism. The role of Gram-negative bacteria may be more relevant in the ICUs. In a meta-analysis of studies investigating the risk of acquiring bacteria from prior bed occupants, the odds ratio for acquisition of *Acinetobacter* was the highest, corresponding to 4.91 (95% CI 2.79–8.64) [5]. The study by Anderson et al. highlights how terminal cleaning may be considered the basis to build an effective strategy to reduce healthcare-associated infections.

## Abbreviations

ICU: Intensive care unit
MDRO: Multidrug resistant organism
UV: Ultraviolet
